# 508 The Effect of Alcohol Use on Fluid Balance During Initial Resuscitation in Burn Patients

**DOI:** 10.1093/jbcr/irae036.143

**Published:** 2024-04-17

**Authors:** Sasha A McEwan, Lori Chrisco, Joyce Pak, Chris B Agala, Leon Coleman, Felicia Williams, booker King, Rabia Nizamani

**Affiliations:** University of North Carolina at Chapel Hill, Chapel Hill, NC; NC Jaycee Burn Center, Chapel Hill, NC; University of North Carolina Chapel Hill, Carrboro, NC; UNC Chapel Hill-School of Medicine, Surgery dept, Chapel Hill, NC; UNC-Chapel Hill School of Medicine, Chapel Hill, NC; University of North Carolina at Chapel Hill, Chapel Hill, NC; NC Jaycee Burn Center, Chapel Hill, NC; University of North Carolina Chapel Hill, Carrboro, NC; UNC Chapel Hill-School of Medicine, Surgery dept, Chapel Hill, NC; UNC-Chapel Hill School of Medicine, Chapel Hill, NC; University of North Carolina at Chapel Hill, Chapel Hill, NC; NC Jaycee Burn Center, Chapel Hill, NC; University of North Carolina Chapel Hill, Carrboro, NC; UNC Chapel Hill-School of Medicine, Surgery dept, Chapel Hill, NC; UNC-Chapel Hill School of Medicine, Chapel Hill, NC; University of North Carolina at Chapel Hill, Chapel Hill, NC; NC Jaycee Burn Center, Chapel Hill, NC; University of North Carolina Chapel Hill, Carrboro, NC; UNC Chapel Hill-School of Medicine, Surgery dept, Chapel Hill, NC; UNC-Chapel Hill School of Medicine, Chapel Hill, NC; University of North Carolina at Chapel Hill, Chapel Hill, NC; NC Jaycee Burn Center, Chapel Hill, NC; University of North Carolina Chapel Hill, Carrboro, NC; UNC Chapel Hill-School of Medicine, Surgery dept, Chapel Hill, NC; UNC-Chapel Hill School of Medicine, Chapel Hill, NC; University of North Carolina at Chapel Hill, Chapel Hill, NC; NC Jaycee Burn Center, Chapel Hill, NC; University of North Carolina Chapel Hill, Carrboro, NC; UNC Chapel Hill-School of Medicine, Surgery dept, Chapel Hill, NC; UNC-Chapel Hill School of Medicine, Chapel Hill, NC; University of North Carolina at Chapel Hill, Chapel Hill, NC; NC Jaycee Burn Center, Chapel Hill, NC; University of North Carolina Chapel Hill, Carrboro, NC; UNC Chapel Hill-School of Medicine, Surgery dept, Chapel Hill, NC; UNC-Chapel Hill School of Medicine, Chapel Hill, NC; University of North Carolina at Chapel Hill, Chapel Hill, NC; NC Jaycee Burn Center, Chapel Hill, NC; University of North Carolina Chapel Hill, Carrboro, NC; UNC Chapel Hill-School of Medicine, Surgery dept, Chapel Hill, NC; UNC-Chapel Hill School of Medicine, Chapel Hill, NC

## Abstract

**Introduction:**

Alcohol use has been shown to have an impact on multiple outcomes during a burn patient’s initial resuscitation and overall hospital course. We have previously described the need for increased fluid resuscitation in the chronic alcohol user. In this study, we aim to demonstrate the difference in fluid balance in the early resuscitation period between chronic alcohol users and non-users, suggesting disordered fluid regulation as a contributor to this discrepancy.

**Methods:**

Using an institutional burn center registry, we identified adult patients admitted from 2017-2020 with total body surface area >10% and a hospital stay >2 days. 298 patients were included in this study and chart review completed for alcohol use status, burn severity, urine output, and fluid and vasopressor administration within 48 hours. Alcohol use was staged using Centers for Disease Control guidelines: none/minimal (NM), early/moderate (EM), and problem/severe (PS). Kruskal-Wallis test was used to assess the difference in the mean fluid balance amongst the three groups. Wilcoxon signed-rank test used to evaluate differences in pairwise comparisons.

**Results:**

Mean fluid balance for each of the groups were compared in ml/kg/TBSA (NM 4.09, EM 2.90, PS 6.87) and a significant difference was demonstrated (Kruskal-Wallis test < 0.001). Wilcoxon signed-rank test of pairwise comparisons between groups demonstrated a significant difference in average fluid balance between each (NM vs. EM, NM vs. PS, EM vs. PS, all < 0.001). Using both the Kruskal test and the Wilcoxon test, there was no significant difference found amongst the average urine output in the three groups, or between any of the pairwise comparisons (Kruskal 0.159; NM vs. EM, 0.265; NM vs. PS, 0.846; EM vs. PS, 0.909). Compared to NM, there was a significant difference in positive blood ethanol test on admission (8.4% increased in EM, 31.4% in PS, p < 0.001 for both).

**Conclusions:**

PS alcohol users had a significantly higher positive fluid balance when compared to NM users. Importantly, there was no significant difference in urine output amongst the three groups or in any pairwise comparison. EM users had a negative fluid balance compared to NM while PS users had a positive fluid balance in the same comparison. Taken together, these results suggest a difference in fluid regulation between different levels of alcohol users. In both the PS and EM groups we found a significant difference in positive blood ethanol tests on admission screen.

**Applicability of Research to Practice:**

We have previously identified decreased serum albumin levels in chronic alcohol users; however, disordered antidiuretic hormone physiology may also contribute to their tendency to third-space during the early resuscitation period. Objective measures of alcohol use, such as blood ethanol level or phosphatidylethanol level, may be useful to help drive resuscitation efforts when determining fluid and pressor type in burn patients.

